# Serious Alert and Border Rejection Notifications on Food in the EU RASFF

**DOI:** 10.3390/vetsci8110279

**Published:** 2021-11-17

**Authors:** Elias P. Papapanagiotou

**Affiliations:** Laboratory of Animal Food Products Hygiene-Veterinary Public Health, Department of Hygiene and Technology of Food of Animal Origin, School of Veterinary Medicine, Faculty of Health Sciences, Aristotle University of Thessaloniki, 54124 Thessaloniki, Greece; ipapapan@vet.auth.gr

**Keywords:** RASFF, serious alert and border rejection notifications, lag phases, FVO, food, Performance Effectiveness Reporting (PER)-50/30, EU Commission, indicator

## Abstract

The serious alert and border rejection notifications on food from the European Union Rapid Alert System for Food and Feed (EU RASFF) database were used to determine their lag phases (from sampling to notification dates). More specifically, 4503 serious alert notifications on food were used to calculate the percent (%) share of various lag phases in an overall fashion (for all EU RASFF Member States collectively examined) as well as for the top-three Member States (in notification numbers), in each one of seven hazard categories. The same procedure was followed for 5236 serious border rejection notifications in each one of five hazard categories on food. The lag phases calculated revealed a state of nonharmonization (in lag phases percent shares) both overall and among the top-three Member States, and in the same MS in various hazard categories in serious alert but less pronounced in serious border rejection notifications. Thus, a “Performance Effectiveness Reporting (PER)-50/30” indicator (over 50% of notifications being notified to the RASFF within 30 days of sampling) was proposed for both types of serious notifications, and its application herein has revealed volatility in performance effectiveness reporting among the top-three EU RASFF Member States in the hazard categories. Actions to harmonize this inconsistency should be pursued in the context of safeguarding public health, aiming at the fastest possible risk management and risk communication of serious contamination incidents on food. Finally, a proposal of an “RASFF country profile” is hereby proposed.

## 1. Introduction

Food safety is a high priority for the EU; it affects all citizens and is closely linked to trade policies. In Article 5 of Regulation (EC) No. 178/2002 (General Food Law), it is stated that the aim is to “guarantee a high level of protection of human life and health” [1]. The Rapid Alert System for Food and Feed (RASFF) of the European Union (EU) was initiated in 1979 and its legal basis is Regulation 178/2002. It is a vigorous tool for the EU Member States to exchange information on risks found in food or feed either entering the EU from a third country or from within the European market. The members of the EU RASFF network are the EU Member States (MS), the European Commission Services, the European Food Safety Authority (EFSA), the countries of the European Economic Area, EEA (Norway, Liechtenstein, Iceland), and Switzerland.

The mode of action of the RASFF is initiated by the notification (be it alert or border rejection that are relevant to the study herein) by a member of the RASFF network of a serious, direct or indirect, risk to public health in food or feed. This information reaches the European Commission (acting as manager of the system), which in turn verifies the notification and immediately transmits it to the other members of the network. A common template is used to provide all relevant and useful information, including identification of the product, hazard(s) found, measure(s) taken and information on tracing the product. Upon receiving the information, other member countries check if they are concerned. If the product is on their market, they are able to trace it using the information in the notification. They report back on what they have found and what measures they have taken for a transparent and mutual information of all RASFF members. In case of products from the EU, the Member State from which the product originates also reports on the outcome of its investigation with regard to the origin, distribution, and cause of the problem identified. This allows other member countries to take rapid action if and when needed.

Among the various types of notifications that have been defined in Regulation (EC) 16/2011 [2], alert notifications are sent when a food or feed (either domestic produce or imports from third countries) presenting a serious health risk is on the market and when rapid action is required. The RASFF member that identifies the problem and takes the relevant actions (e.g., withdrawal of the product) triggers the alert. The goal of the notification is to give all RASFF members the information to confirm whether the product in question is on their market, so that they can also take the necessary measures. The border rejection notifications concern food and feed consignments that have been tested and rejected at the external borders of the EU and the European Economic Area (EEA) when a health risk has been found. The notifications are sent to all EEA border posts in order to reinforce controls and to ensure that the rejected product does not re-enter the EU through another border post. A significant improvement in the EU RASFF Portal database has been the introduction of a relatively new category namely “risk decision” which is effectively analyzed into three options, namely “serious”, “not serious”, and “undecided”. The “serious” notifications and, more specifically, the alert and border rejection notifications that have been assigned to this category are the target of analysis for the first time in bibliography for food, in this paper. None of the publications on the EU RASFF data on food have focused so far on the risk-designated “serious” notifications originating from the RASFF Portal database. The “serious” risk decision is existing and available for RASFF notifications from 2011 (18 November 2011) for border rejection notifications and from 2012 (27 June 2012) for alert notifications.

The Commission publishes a searchable online database of RASFF notifications classified as alert, information notification, or border rejection as part of its RASFF Portal. The various options in the RASFF database for the notification basis, indicating what type of control, report, or investigation lay at the basis of the notification, is the following: (i) border control—consignment released (notification initiated through a sample taken at a border post for analysis but the consignment was meanwhile released to the market), (ii) official control on the market (official control on the EEA internal market), (iii) company own-check (notification initiated through a company notifying the outcome of an own-check to the competent authority), (iv) consumer complaint (notification initiated through a consumer lodging a complaint with the competent authority), (v) food poisoning (reports of a food poisoning leading to the notification of a risk in a food on the market that has caused the food poisoning). The 2019 RASFF Annual Report [3] specified that in 2019, 40% of RASFF notifications concerned controls at the outer EEA borders, at points of entry or border control posts. In other cases, the consignment was released (“border control—consignment released”) without awaiting the analytical result, which means that the consignment would need to be retraced if the result was unfavorable and the product needed to be withdrawn from the market. Therefore, the latter cases lead to alert or information notifications. A small number of notifications were triggered by an official control in a nonmember country.

Another EU institution that is closely related to the RASFF is the Health and Food Audits and Analysis or ex-Food and Veterinary Office, FVO of the European Commission. While the role of the FVO does not include early warning activities, the inadequacies of food safety control in different countries subject to FVO audit could be used as an indicator/early warning of future food safety problems, and the FVO recommendations could be used as preventative measures which include recommendations to national authorities but also to the European Commission on legislative changes that may be required to prevent or reduce the likelihood of foodborne illness in the future through European food law [4].

The Official Food and Feed Controls (OFFC) Regulation (EC) No 882/2004 forms the basis for the checks carried out [5]. The OFFC Regulation aimed at an integrated and uniform approach to official controls along the agri-food chain. It provided the framework for competent authorities to verify compliance with food and feed law and to prevent, eliminate, and reduce, to acceptable levels, risks to human beings and animals. The abovementioned OFFC Regulation has recently been replaced by The Official Controls Regulation, OCR (EU) 2017/625, which addresses official controls and other official activities performed to ensure the application of food and feed law, rules on animal health and welfare, plant health, and plant protection products, was published in the Official Journal of the European Union on 7 April 2017 and entered into force on the 27 April 2017 [6].

An interesting publication [7] has discussed the challenges of interpretation and acting upon the dataset contained within the RASFF with specific emphasis on mycotoxin contamination.

Furthermore, the actual yearly RASFF Annual Reports have only initiated presenting data on notifications by hazard category and risk decision in 2015 [8], in 2016 [9], in 2017 [10], in 2018 [11], and in 2019 [3]. Alas, the only information given is the number of notifications (all types) that have been designated to one of the three categories, namely “undecided”, “serious”, and “not serious”. Nevertheless, a wealth of invaluable information could be extracted from looking closer to “serious” notifications and, more specifically, the “serious” alert and the border rejection notifications, as was attempted herein for food.

The aim of this paper was fivefold: (i) firstly, to calculate lag phases (from sampling to notification dates) of serious alert notifications as percent (%) shares in an overall fashion, incorporating all RASFF EU MS for seven hazard categories in food, namely, mycotoxins, microbial contaminants (other), foreign bodies, composition, allergens, metals, and pathogenic microorganisms (PMO) from the RASFF database, (ii) secondly, to employ the same rationale for serious border rejection notifications for all RASFF EU MS for five hazard categories in food, namely, mycotoxins, microbial contaminants (other), pesticide residues, metals, and pathogenic microorganisms (PMO) from the RASFF database, (iii) thirdly, to employ the same pattern of calculation of lag phases for the top-three Member States (in numbers of each type of notification) for all the hazard categories in food examined in both types of serious notifications, (iv) fourthly, to institutionalize the first performance effectiveness reporting indicator in order to successfully gauge the RASFF EU MS’s performance effectiveness reporting of serious alert and serious border rejection notifications, and (v) finally, to introduce proposals to the EU Commission concerning a roadmap towards a harmonized pattern of timely notification effectiveness in food, based on the lag phases percent (%) shares volatility.

In essence, this work has focused solely on “lag phases”, meaning the time interval (in days) from sampling to notification dates (as these appear in the details of the serious alert and serious border rejection notifications examined herein), and by further utilizing these, on introducing a performance effectiveness reporting indicator (PER-50/30) within the context of the EU RASFF.

## 2. Materials and Methods

All the data used in this paper were retrieved from the RASFF Portal (https://webgate.ec.europa.eu/rasffwindow/portal/?event=SearchForm&cleanSearch=1 (accessed on 16 May 2020) [12]. The EU RASFF database was used in order to find the sampling date and the notification date of serious alert (sA) notifications (from the details of each notification) on food, in seven heavily notified hazard categories, which are representative of all three hazard types (biological, physical, and chemical) as defined by the Codex Alimentarius Commission of the FAO/WHO [13]. Seven food hazard categories have been selected to be examined, namely, mycotoxins, microbial contaminants (other), foreign bodies, composition, allergens, metals, and pathogenic microorganisms (PMO) from the RASFF database; in this paper, the reason being that the sum of the sA notifications of these hazard categories represent, overall, collectively (from 1 January 2012 until 31 December 2019), more than 82% of all sA notifications of all hazard categories in the EU RASFF database. The hazard category “foreign bodies” refers to physical hazards (e.g., glass, metal plastic fragments, etc.), whereas the hazard category “composition” refers to hazards such as undeclared substances or unauthorized color, ingredients, etc.). The hazard category “microbial contaminants” used to be the hazard category “nonpathogenic organisms” in the RASFF Portal database.

By the same token, serious border rejection (sBR) notifications allocated to five food hazard categories representing over 93% of all such notifications submitted to the EU RASFF were examined (from 1 January 2011 until 31 December 2019).

The rationale behind the search of the overall lag phases (from sampling to notification dates) was effectively employed in the top-three Member States (in numbers of sA and sBR notifications submitted to the EU RASFF) in all hazard categories examined in this paper.

Searches were performed in the EU RASFF Portal database (https://webgate.ec.europa.eu/rasff-window/portal/, (accessed on 16 May 2020)) by using the following key search terms used in this study: (i) Risk Decision “serious” AND Type “food” AND hazard categories “mycotoxins; microbial contaminants (other); foreign bodies; composition; allergens; metals; pathogenic microorganisms” AND “alert notifications” and (ii) Risk Decision “serious” AND Type “food” AND hazard categories “mycotoxins; microbial contaminants (other); pesticide residues; metals; pathogenic microorganisms” AND “border rejection notifications”

From the RASFF Portal, the notification basis (border control—consignment detained (BCCD), border control—consignment released (BCCR), company’s own check (COC), food poisoning (FP), monitoring of media (MoM) which mainly points to monitoring of products sold online, official control in non-member state (OCnonMS), official control on the market (OCTM)) was also retrieved and used in this study for each of the food hazard categories in serious alert notifications.

A subtraction (notification dates minus sampling dates) was calculated, and the time interval was expressed in days. Then, a group categorization was made, namely, 0–10 days, 11–20 days, 21–30 days, 31–40 days, 41–50 days, 51–100 days, ≥101 days. The sA and sBR notifications falling in each lag phase were expressed as a relative percent (%) share of all such notifications, respectively.

Moreover, shortcomings were found concerning both the sA and sBR notifications when (i) no sampling date was given in the EU RASFF database (from the details of each notification); this was reported as “no sampling date”, (ii) an obviously irregular date (e.g., 1/1/1970) appeared in the relevant section; this was reported as “dated 1/1/1970”, and (iii) the dates of notification and sampling were clearly inverse (when the stated notification date was earlier than the sampling date); this was reported as “inverse dates”. For these notifications that had these shortcomings, lag phases could not be calculated, and these composed a sum (as a relative percent share), namely “Subtotal 2”.

## 3. Results

### 3.1. Serious Alert (sA) Notifications in Seven Hazard Categories on Food (2012–2019) from the EU RASFF Database

The first sA notification ever to be submitted in the RASFF database was by Sweden on 27 June 2012 on “dietetic foods, food supplements, fortified foods”, its subject being “unauthorized substance 1,3 dimethylamylamine (DMAA) in food supplement from the United States”. The sA notifications submitted in the EU RASFF database for the seven hazard categories analyzed herein, on a yearly basis, are shown in Table 1. The overall percent (%) share of the sA notifications for all seven hazard categories ranges from 78.1% to 84.9%.

The overall sA notification incidence of the hazard categories under examination, in descending order, was as follows: pathogenic microorganisms (PMO) > allergens > microbial contaminants (other) > metals > mycotoxins > composition > foreign bodies.

#### 3.1.1. The Notification Basis of sA Notifications Submitted for the Seven Hazard Categories on Food, for All EU RASFF MSs Collectively (Overall) from the EU RASFF Database (2012–2019)

The allocation of sA notifications submitted for the selected seven hazard categories on the basis of OCTM, COC, CC, FP, and MoM (monitoring of media), is shown in Table 2. Of interest is the classification of the >90% share of the sA notifications, made on the basis of the most frequently found reason, in all the hazard categories examined.

In more detail, in metals (93.3%), microbial contaminants (other) (94.7%), and mycotoxins (93.4%), the combined contribution of OCTM and COC exceeded 90%, whereas in PMO, it was the combined contribution of OCTM, COC, and FP that cumulatively surpassed that percentage (97.1%). In composition and foreign bodies, that percentage was exceeded by the combined contribution of OCTM, CC, and MoM for the former (93.4%), and COC and CC for the latter (95.9%). Finally, in allergens, the 90% cutoff point of all sA examined herein was exceeded (93.8%) by the combined contribution of OCTM, COC, and CC.

Additionally, the percent (%) share of sA notifications examined in this work that was made on the basis of “border control—consignment released” or BCCR on mycotoxins (5.79%) and metals (5.85%) was particularly interesting and is thus highlighted. This is a case in which the consignment was released without awaiting the analytical result, which means that the consignment would need to be retraced if the result was unfavorable and the product needed to be withdrawn from the market. Therefore, these cases lead to alert or information notifications.

Interestingly enough, the range of the percent (%) share of OCTM and of COC in the different hazard categories was quite broad, namely 4.1–83.2% and 2.7–53.7%, respectively. Another finding of interest was the high FP representation in PMO only to be seconded in ranking by allergens. The MoM was only represented in composition and no other hazard category. Another striking finding was revealed for CC in foreign bodies with a rather too-high percent (%) share.

#### 3.1.2. Lag Phases (from Sampling Dates to Notification Dates) Criterion Applied in sA Notifications on Food, for All EU RASFF MSs Collectively (Overall)

The subtraction of sampling dates from notification dates in the 4503 sA notifications examined herein produced the lag phases for the seven selected food hazard categories for all EU RASFF MS collectively, which are categorized into groups of days (e.g., 0–10 days, 11–20 days, 21–30 days, etc.) for a more comprehensive presentation of the rather large dataset, as shown in Table 3.

The various hazard categories examined herein exhibit their maximum and minimum percent (%) shares in different lag phases within Subtotal 1 (more specifically, metals (0–10 days and ≥101 days), microbial contaminants (other) (0–10 days and ≥101 days), mycotoxins (51–100 days and ≥101 days), PMO (11–20 days and ≥101 days)). For the remaining three hazard categories, namely, composition, foreign bodies, and allergens, the high percent share of Subtotal 2 (32.4%, 82.6%, and 59.4%, respectively) would render the designation of maximum and minimum percent shares of no avail.

The overall picture is quite diverse with lag phases percent (%) shares’ distribution of sA notifications depending on the hazard category. For example, the ranking of the hazard categories whose sA notifications were allocated in the lag phase of 0–10 days is the following (in descending order): microbial contaminants (other) > metals > pathogenic microorganisms (PMO) > foreign bodies > mycotoxins > allergens > composition. This lag phase expressed as a percent (%) share of all such notifications within each hazard category ranges widely between all seven hazard categories (from 7.2% to 43.8%). A similar finding was revealed for other lag phases percent (%) shares, e.g., the 11–20 days lag phase (3.0–31.7%), the 21–30 days (0.7–15.7%), etc.

The fact that the EU RASFF MSs can indeed (to a rather varied degree as witnessed above) submit a sA notification within 10 days from sampling is noteworthy, although this finding does not hold true throughout all seven hazard categories, indicating a state of nonuniformity across the board.

Furthermore, in defense of the aforementioned highly promising finding, examples of sA notifications that have the same notification and sampling date (meaning a zero lag phase) have been identified, and these are the following, submitted by (i) the Netherlands on 21 March 2018, its subject being *Listeria monocytogenes* in raw milk brie cheese from France (microbial contaminants (other), COC), (ii) Denmark on 31 January 2013, its subject being undeclared celery in hot chicken wings from Denmark (allergens, COC), (iii) Belgium on 17 May 2013, its subject being *Salmonella enterica* in salami without garlic from Belgium (PMO, COC), (iv) Italy on 9 August 2013, its subject being mercury in chilled shortfin mako shark (*Isurus oxyrhinchus*) from Spain (metals, OCTM), and (v) France on 19 December 2019, its subject being lead in green clay from France (metals, COC).

On the other hand, the lag phase of ≥101 days, which ranges from 0 to 11.7% between the seven hazard categories, depends on the hazard category and, in descending order, the ranking is the following: composition > mycotoxins > metals > allergens > pathogenic microorganisms (PMO) > microbial contaminants (other) > foreign bodies.

The ranking for the hazard categories examined (in descending order) vis a vis Subtotal 2 (encompassing all shortcomings of the notifications) was found to be the following: foreign bodies > allergens > composition > pathogenic microorganisms (PMO) > microbial contaminants (other) > mycotoxins > metals.

On the other hand, the hazard categories that exhibited the smallest percent shares in Subtotal 2 were mycotoxins and metals. Subtotal 2, once again, depending on the hazard category, ranges from 1.9% to 82.6%.

One truly interesting result originating from Table 3 is the specific lag phase exhibiting the highest percent (the most numerous sA notifications allocated in it) for each of the seven hazard categories. For example, in mycotoxins, the highest percent (%) share of sA notifications was allocated in the lag phase of 51–100 days. Furthermore, in composition, in foreign bodies and in allergens, the vast majority of such notifications were allocated under “no sampling date”. Lastly, in both metals and microbial contaminants (other), and exclusively in pathogenic microorganisms (PMO), the highest percent (%) share of such notifications fell in the lag phase of 0–10 days and 11–20 days, respectively.

In a more detailed view, the percent (%) share of the sA notifications of each one of the hazard categories examined herein that fell into the lag phase of 0–30 days (found by adding the respective percent shares of the three lag phases, namely, 0–10 days, 11–20 days, and 21–30 days), was 69.8% in metals, 84.9% in microbial contaminants (other), 40.1% in mycotoxins, 71.7% in pathogenic microorganisms (PMO), 20.9 % in composition, 16.2% in foreign bodies, and 22.3% in allergens.

Interesting conclusions can also be revealed, for example, if one would set a cutoff point in the lag phase of 0–30 days as a reasonable response time, with a prerequisite of an attained percent (%) share of at least 50% of sA notifications falling in that lag phase. More specifically, the hazard categories that would comply with this theoretical exercise would only be microbial contaminants (other), PMO, and metals. The hazard category mycotoxins was close enough with 40.1%, while all of the remaining hazard categories (composition, allergens, and most distinctly foreign bodies) would not comply with this theoretical (but reasonable) criterion. One should always bear in mind that the aforementioned hazard categories exhibited too-high percent (%) shares of Subtotal 2 (shortcomings as a whole), which would inevitably render them incapable of complying with the cutoff point, as explained before.

#### 3.1.3. Lag Phases (from Sampling Dates to Notification Dates) Criterion Applied in sA Notifications on Food, for the Top-Three EU RASFF MSs

The average percent share of the 0–30 days lag phase of the top-three MSs in mycotoxins was 44.9% (range 19.7–84.9%), as shown in Table 4. The average percent share of the 0–30 days lag phase of the top-three MSs in metals (range 38.4–88.8%), microbial contaminants (other), (range 88.9–91.6%), and PMO (range 52.3–76.2%) was 57.3%, 90.3%, and 65.7%, respectively.

The Netherlands (NL) is the only MS with a place in the top-three contributing notifying countries in five out of seven hazard categories. The NL has performed quite well in certain hazard categories, e.g., metals, since the highest percent (25.6%) share of its sA notifications was reported in the 11–20 days lag phase. Finally, the NL had a cumulative percent (%) share of the lag phase 51–≥101 days of 69.7%, in mycotoxins, whereas it was only 6.6% in PMO.

Germany (DE) is another interesting example of a top-three EU RASFF MSs being present in four out of seven hazard categories examined herein. In metals, DE had the highest attained percent share of all its sA in the 0–10 days lag phase. Finally, DE had a cumulative percent (%) share of the lag phase 51–≥101 days of 41.4% and 40.0% in metals and mycotoxins, respectively.

Italy (IT) was present in the top-three ranking MSs in three out of seven hazard categories examined herein, showing the highest attained percent (%) share of all its sA in metals, in PMO, and in microbial contaminants (other), in the 0–10 days lag phase.

France (FR) appeared in the top-three EU RASFF MSs in three hazard categories, namely composition, PMO, and microbial contaminants (other). With regards to composition, its highest percent (%) share of all sA was in the 51–100 days lag phase, in PMO in the 11–20 days lag phase, and in microbial contaminants (other) in the 0–10 days lag phase.

Belgium (BE) appeared in the top-three EU RASFF MSs in three out of seven hazard categories, namely, mycotoxins, allergens, and microbial contaminants (other). Its highest percent (%) shares of its sA in microbial contaminants (other) and mycotoxins were in the 0–10 days and the 11–20 days, respectively.

The United Kingdom (GB) appeared in the top-three EU RASFF MSs in two (foreign bodies and allergens) out of seven hazard categories with a Subtotal 2 percent share being 100.0% and 80.8% of all its sA notifications, respectively.

Sweden (SE) was found in the top-three EU RASFF MSs only in one out of seven hazard categories, namely composition. A total of 31.1% was allocated to the 0–10 days lag phase but 50.8% was allocated to the “no sampling date” shortcoming.

#### 3.1.4. The Notification Basis of sA Notifications Submitted for the Seven Hazard Categories on Food, for the Top-Three EU RASFF MSs from the EU RASFF Database (2012–2019)

As can be seen in Table 5, the vast majority (>90% taken as a cutoff point) of sA notifications were submitted on the basis of OCTM alone in IT, of OCTM and COC in the NL, and of OCTM and COC and BCCR in DE, with OCTM prevailing (in percent share) in metals. In microbial contaminants (other), the combined contribution of OCTM and COC was revealed, with COC prevailing over OCTM in FR and BE. In mycotoxins, the combined contribution of OCTM and COC was evidenced for DE and the NL (with OCTM prevailing), whereas in BE it was the combined contribution of OCTM, COC, and BCCR (with COC prevailing). In PMO, it was COC in FR and the NL that prevailed, and the combined contribution of OCTM, COC, and FP attained the >90% of sA notifications, whereas in IT it was OCTM that prevailed, and the combined contribution of OCTM and FP exceeded the 90% cutoff point. In composition, in the cases of DE and FR, OCTM prevailed (alone in the latter) and with the combined contribution of CC in the former, exceeded the >90% cutoff point, whereas in SE, the combined contribution of MoM (that prevailed) with OCTM exceeded the >90% cutoff point. In foreign bodies, the combined contribution of COC and CC attained the >90% cutoff point with COC prevailing in GB and the NL, whereas in DE, it was CC that prevailed. In allergens, COC prevailed and the combined contribution of OCTM and COC for GB and BE exceeded the >90% cutoff point, whereas for the NL, the combined contribution of COC and CC attained the >90% cutoff point.

In this exercise, one can interconnect the volatility in response time (performance effectiveness reporting) from country to country according to the percent (%) share of the basis that their sA notifications have been made on, comparatively. Thus, one can compare each of the top-three EU RASFF MSs against the overall values shown in Table 2 where all MSs’ contributions have been collectively reported.

More specifically, in metals, for BCCR, the overall cutoff point (5.8%) was largely surpassed by DE but not by IT and the NL. In microbial contaminants (other), the overall cutoff point (53.7%) for COC was surpassed by FR and BE but not by IT. In mycotoxins, the overall cutoff point (72.1%) for OCTM was surpassed by DE and the NL but not by BE. Finally, for BCCR, the overall cutoff point (5.8%) was largely surpassed by BE but not by DE and the NL. In PMO, the overall cutoff point (48.3%) for COC was surpassed by FR and the NL but not by IT. However, IT and FR surpassed the overall cutoff point of 12.3% in FP but not the NL. In composition, the overall cutoff point (72.7%) for OCTM was surpassed by DE and FR but not by SE, whereas SE surpassed, by far, the cutoff point of 15.8% in MoM. In foreign bodies, the overall cutoff point (63.5%) for CC was exceeded only by DE but not by the NL and GB. In allergens, the overall cutoff point (47.5%) for COC was surpassed by all top-three MS (GB, NL, BE); however, none of the top-three MS exceeded the cutoff point of 10.3% in CC.

#### 3.1.5. The Performance Effectiveness Reporting-50/30 (PER-50/30) as a Proposed Indicator of Effectiveness for the Top-Three EU RASFF MSs in sA Notifications in Seven Hazard Categories on Food

A state of lack of harmonization in the percent (%) shares of lag phases’ (from sampling to notification dates) allocation of sA notifications was revealed among the top-three EU RASFF MSs (according to their number of sA notifications contributed to the RASFF for each of the seven hazard categories examined). Thus, a proposed “Performance Effectiveness Reporting-50/30” (PER-50/30) indicator, signifying a threshold limit of timely reporting of sA (in the EU RASFF) for the top-three EU RASFF MS, was further examined. In more detail, if a cutoff limit of at least 50% of sA notifications being notified to RASFF within the lag phase of 0–30 days (originating from the sum of the percent shares of 0–10 days, 11–20 days, and 21–30 days lag phases) from sampling were to be employed, the MSs that would satisfy this indicator would be the following:

1. Metals: IT.
2. Microbial contaminants (other): all three MSs (FR, IT, NL).
3. Mycotoxins: BE.
4. PMO: all three MSs (FR, NL, IT).
5. Composition: all three MSs (DE, SE, FR).
6. Foreign bodies: all three MSs (DE, GB, NL).
7. Allergens: all three MSs (GB, NL, BE).

What is clear from the results is that microbial contaminants (other) and PMO are hazard categories for which a PER-50/30 criterion could easily be complied with (and thus justified) for use in the top-three EU RASFF MSs. On the other hand, a possible introduction of a PER-50/30 in metals and mycotoxins would be easily achieved by only one of the top-three EU RASFF MSs. Concerning three categories (composition, foreign bodies, and allergens) the shortcomings revealed herein (too-high Subtotal 2) would not allow for the calculation of lag phases, so one could not estimate how the top-three EU RASFF MSs would have performed.

### 3.2. Serious Border Rejection (sBR) Notifications in Five Hazard Categories on Food (2011–2019) from the EU RASFF Database

The first sBR notification to be submitted in the RASFF database was by the Netherlands (Reference number 2011.CKE) on 18 November 2011 on herbs and spices and it was a BCCD (border control—consignment detained), its subject being “*Salmonella* (1 out of 5 samples/25 g) in rau ram leaves/*Polygonum odoratum* (praew leaves) from Vietnam”. The sBR notifications submitted in the EU RASFF database on a yearly basis for the five hazard categories selected to be analyzed herein are shown in Table 6.

The overall notification frequency of the sBR notifications on food, in the five hazard categories in descending order, was as follows: mycotoxins > PMO > pesticide residues > microbial contaminants (other) > metals.

#### 3.2.1. Lag Phases (from Sampling Dates to Notification Dates) Criterion Applied in sBR Notifications on Food, for All EU RASFF MSs Collectively (Overall)

The subtraction of sampling from notification dates in the 5593 sBR notifications examined herein produced the lag phases of the five selected food hazard categories for all EU RASFF MSs collectively, which are categorized into groups of days (e.g., 0–10 days, 11–20 days, 21–30 days, etc.), as shown in Table 7.

The various hazard categories examined herein exhibit their maximum and minimum percent (%) shares in different lag phases within Subtotal 1, more specifically, metals (0–10 days, 11–20 days and ≥101 days), microbial contaminants (other) (0–10 days and ≥101 days), mycotoxins (11–20 days and ≥101 days), PMO (11–20 days and ≥101 days), and pesticide residues (0–10 days and ≥101 days).

The lag phases’ distribution of sBR notifications depends on the hazard category. More specifically, the ranking of the five hazard categories whose sBR notifications were mostly assigned in the lag phase of 0–10 days was the following (in descending order): pesticide residues > metals > microbial contaminants (other) > PMO > mycotoxins. This lag phase expressed as a percent (%) share of all such notifications within each hazard category ranges, between the five hazard categories, from 16.6% to 60.4%. A similar finding was revealed for other lag phases percent (%) shares, e.g., the 11–20 days lag phase (13.8–43.9%), etc.

Evidently, the EU RASFF MSs have proved (to a rather varied degree, as witnessed above) their capacity to submit sBR notifications within 10 days from sampling which is very important, besides the fact that some inconsistency in the 0–10 days lag phase between all hazard categories does exist.

Some examples (certainly more numerous than those in sA notifications) of zero days lag phase have been found and are as follows: (i) all were BCCD and submitted by Bulgaria on pesticide residues (Notification Reference numbers: 2019.4208, 2019.3857, 2019.0457, 2018.3734, 2018.3293, 2018.3006, 2018.0212, 2018.0126, 2017.CHO, 2017.BXN, 2017.BWM, 2017.BSV, 2017.BON, 2017.BLU, 2017.BCM, 2017.AHV, 2017.AHO, 2017.AEX, 2017.AFC, 2017.ADZ, 2017.0042, 2016.BQF, 2016.BOJ, 2016.BKK, 2016.BDL, 2016.BDA, 2016.AXA, 2016.ANE, 2016.ALZ, 2016.AJD, 2015.BTT, 2015.AKX, 2015.AKS, 2014.BHG, 2014.ALA, 2013.BXW), (ii) all were BCCD and submitted by the Netherlands on microbial contaminants (other) (Notification Reference numbers: 2017.BAC, 2017.AVY), and (iii) all were BCCD and submitted by Denmark, Austria, and Italy on mycotoxins, respectively (Notification Reference numbers: 2016.AHN, 2015.BPT, 2013.ANC).

Conversely, the lag phase of ≥101 days ranges from 0.6 to 5.3% and also depends on the hazard categories; the ranking of the hazard categories, in descending order, is the following: mycotoxins > pesticide residues > PMO > metals > microbial contaminants (other).

Subtotal 2 was calculated to be, in the hazard categories examined (in descending order), the following: microbial contaminants (other) > pesticide residues > mycotoxins > PMO > metals. Subtotal (2), depending on the hazard categories, ranges from 2.0% to 10.2%.

The percent (%) share of the sBR notifications of each one of the hazard categories examined herein that fall into the lag phase of 0–30 days (found by adding up 0–10 days, 11–20 days, and 21–30 days respective percent shares), in metals was 81.6%, in microbial contaminants (other) was 69.2%, in mycotoxins was 63.3%, in PMO was 82.1%, and in pesticide residues was 80.7%.

#### 3.2.2. Lag Phases (from Sampling Dates to Notification Dates) Criterion Applied in sBR Notifications on Food, for the Top-Three EU RASFF MSs

The percent (%) distribution of lag phases concerning sBR notifications of the top-three EU RASFF MS in the five hazard categories examined on food are shown in Table 8.

The widest range between the top-three MSs was noticed in mycotoxins, where the highest and lowest value was allocated to GB and the NL, respectively, whereas DE exhibited an intermediate value of 66.36%. The average percent share of the 0–30 days lag phase of the top-three MS in mycotoxins was 61.4% (range 45.6–72.3%). The average percent share of the 0–30 days lag phase of the top-three MS in metals (range 85.7–89.5%), microbial contaminants (other) (range 66.6–84.6%), and PMO (range 73.7–89.4%) was 88.0%, 77.8%, and 82.4%, respectively.

The Netherlands (NL) is the MS with a place in the top-three notifying countries in all but two hazard categories, namely, metals and pesticide residues. In more detail, its cumulative percent (%) share of sBR allocated in the lag phase of 50–≥101 days was 35.671%.

The United Kingdom (GB) appears as a top-three EU RASFF MSs in three (mycotoxins, PMO, and pesticide residues) hazard categories, with a cumulative percent (%) share of the lag phase 51–≥101 days in mycotoxins of 13.7%, 11.3% in pesticide residues, and 4.1% in PMO.

Germany (DE) is another interesting example of a top-three EU RASFF MSs being present in all but three (PMO, pesticide residues, and metals) hazard categories examined. In mycotoxins, DE had a cumulative percent (%) share of the lag phase 51–≥101 days of 13.4%, and zero in microbial contaminants (other).

Italy (IT) was present in the top-three ranking MSs in two, namely, metals and microbial contaminants (other), hazard categories examined, showing a cumulative percent (%) share of the lag phase 51–≥101 days of 2.8% in metals and of 3.8% in microbial contaminants (other).

France (FR) appeared in the top-three EU RASFF MSs only in two out the five hazard categories, namely, metals and pesticide residues. The cumulative percent (%) share of the lag phase 51–≥101 days was 5.2% in metals, while in pesticide residues it was 31.1%.

Spain (ES) appeared in the top-three EU RASFF MSs in one hazard category, namely, metals. Its contribution was shown to have a cumulative percent (%) share of the lag phase 51–≥101 days, a remarkable zero percent.

Greece (GR) was present in one of the hazard categories examined as a top-three EU RASFF MSs, and this was PMO. The cumulative percent (%) share of the lag phase 51–≥101 days was 13.8%.

Bulgaria (BG) was found in the top-three EU RASFF MSs only in one hazard category, namely, pesticide residues. An impressive 82.3 % of its sBR was allocated to the 0–10 days lag phase and, furthermore, a mere 1.6% was allocated to the cumulative percent (%) share of the lag phase 51–≥101 days.

If one were to set a cutoff point in the lag phase of 0–30 days as a reasonable response time, with a prerequisite of an attained percent (%) share of at least 50% of sBR notifications falling in that lag phase, all the hazard categories would comply with this theoretical exercise.

#### 3.2.3. The Performance Effectiveness Reporting-50/30 (PER-50/30) as a Proposed Indicator of Effectiveness for the Top-Three EU RASFF MS in sBR Notifications in Five Hazard Categories on Food

If a proposed “Performance Effectiveness Reporting-50/30” indicator, representing a threshold limit for the top-three EU RASFF MSs, were to be examined (e.g., a cutoff limit of 50% of sBR notifications being notified to RASFF within the lag phase of 0–30 days from sampling), the MSs that would satisfy this indicator would be the following:

1. Metals: all three MSs (FR, IT, ES).
2. Microbial contaminants (other): all three MSs (NL, IT, DE).
3. Mycotoxins: DE and GB.
4. Pathogenic microorganisms: all three MSs (NL, GB, GR).
5. Pesticide residues: BG and GB.

The findings show that microbial contaminants (other), metals, and PMO are hazard categories that a PER-50/30 criterion could easily be employed, and consequently justified, for use in the top-three EU RASFF MSs. On the other hand, a possible introduction of a PER-50/30 in pesticide residues and mycotoxins would be easily satisfied by two of the top-three EU RASFF MSs. The top-three EU RASFF MSs seem to have performed quite successfully when it comes to sBR notifications in all five of the hazard categories examined here (with only a few exceptions).

### 3.3. Insight into the Top-Three EU RASFF MS Appearing Both in sA and sBR Notifications in the Four Common Hazard Categories Examined (Metals, Microbial Contaminants (Other), Mycotoxins, and PMO) on Food

Of the four common (in both sA and sBR notifications) food hazard categories examined herein (metals, microbial contaminants (other), mycotoxins, and PMO), the frequency of appearance of the top-three EU MSs was the following (in descending order) in sA notifications: NL and IT (*n* = 3 each) > DE, BE, and FR (*n* = 2 each), and in sBR notifications: NL (*n* = 3) > GB, DE, and IT (*n* = 2 each) > FR, ES, and GR (*n* = 1 each), respectively. Furthermore, the combinations of the top-three EU RASFF MSs that appeared in both sA and sBR in the same specific hazard categories were the following five: IT in metals, IT in microbial contaminants (other), DE and NL in mycotoxins, and, finally, NL in PMO.

The effectiveness of each of the abovementioned EU MSs can be well illustrated when one compares the lag phases of the sA and sBR notifications that these MS have reported. The five case studies, when examined individually, could indicate possible areas worth highlighting for future reference.

The case studies of IT with metals and IT with microbial contaminants (other) do not seem to reveal worth mentioning differences between the sA and sBR timely notifications effectiveness vis a vis their allocation into lag phases.

On the other hand, the same does not hold true for the remaining three case studies. More specifically, these are rather quite challenging when one compares the cumulative percent share of the lag phases of 0–50 days and 51–≥101 days.

In the case study of DE and mycotoxins, the respective values for the 51–≥101 days lag phases were 37.50% for sA, while for sBR were only 9.55%. The sA notifications exceeded 3.4 times the percent share of the 11–20 days lag phase in comparison with the sBR, and 3.9 times the percent share of the 51–100 days lag phase.

In the case study of NL and mycotoxins, the respective values for the ≥101 days lag phases were 33.33% for sA, while for sBR, were only 15.63%. The sA notifications was zero in the percent share of the 0–10 days lag phase, in comparison with the sBR, which was 9.62%. In the case study of NL and PMO, the respective values for the 0–50 days cumulative lag phases were 57.61% for sA, while for sBR were 93.18%, and Subtotal 2 for sA and sBR were 35.76% and 1.95%, respectively.

It is worth mentioning that the group of the top-three MSs in sA notifications for the four hazard categories examined here consisted of only five countries, namely, IT, NL, DE, FR, and BE, whereas in sBR, notifications consisted of seven countries, namely, NL, IT, DE, GB, FR, GR, and ES.

Of course, the rationale described above could be easily applied to each one of the EU RASFF MSs (not only to the top-three) in order to find differences in their effectiveness in notifying sA or sBR notifications.

Differences noted when comparing the same EU MS in its effectiveness to timely submit the two different types (sA and sBR) of notifications could be further pursued towards each MS’s effectiveness to report on specific hazards (e.g., *Salmonella* spp., *Escherichia coli*, aflatoxins, mercury, cadmium, etc.). In addition, these differences (if present) could be thoroughly examined on a yearly basis and thus following closely the reporting history of each EU MS concerning lag phases (sampling dates to notification dates) and the PER-50/30 indicator by correlating it with possible changes in control patterns, sampling frequencies, etc.

## 4. Discussion

The hazard categories that consistently had the highest overall relative percent (%) share in sA and sBR notifications in the hazard categories on food examined herein were PMO and mycotoxins with 26.3% and 53.1%, respectively. Moreover, in the sA notifications, the third-ranked hazard category was microbial contaminants (other) with 15.7%, a fact that clearly points to the fact that microbial hazards, as a whole, with a collective percent share of 42.0% over the 8-year period, dominate the picture. It becomes quite self-evident that the overall microbial hazards for the sA and the chemical hazards (mycotoxins) for the sBR should rightfully be the center of attention for any improvement targeting the MS’s performance effectiveness reporting in the EU RASFF.

All the stakeholders involved in safeguarding food safety, and thus public health, representing three pillars (authorities, industry, and consumers) bearing the shared responsibility for a safe product offered to the consumer, were represented in various relative percent (%) shares in the sA notifications, depending on the hazard category examined. Overall (for all EU RASFF MS), the majority of sA notifications in metals, mycotoxins, and composition were submitted on the basis of OCTM (authorities), whereas in microbial contaminants (other), PMO, and allergens, on the basis of COC (industry). Only in foreign bodies was the majority of sA notifications made on the basis of CC (consumers).

The sA notifications of the combined overall microbial hazards (PMO and microbial contaminants (other)) for all EU MS collectively examined were made on the basis of COC and OCTM in the vast majority of cases. The same was evident for mycotoxins (chemical hazard) with COC and OCTM.

Through a more detailed investigation of the large datasets examined herein, differences between the top-three notifying countries in all seven hazard categories were revealed. Examples that stand out are: (i) Sweden, who has submitted the majority of its sA notifications in composition on the basis of MoM, and (ii) Belgium and Germany, who have submitted the majority of their sA notifications on mycotoxins and metals on the basis of BCCR. This individual (each EU RASFF MS) picture that is depicted herein possibly reflects not only each country’s strategic planning over the years examined for sampling and analysis of food, but also its culture on the part of its consumers. Thus, the differences on the basis of sA notifications being submitted to the EU RASFF could be explained through the individual characteristics that exist in each country, signifying each country’s drive to strengthen each of the three pillars associated with the basis of sA notifications that are being submitted to the EU RASFF.

Furthermore, a number of shortcomings (collectively addressed as Subtotal 2) in the effectiveness of submitting sA and sBR notifications were revealed in this study. With regards to Subtotal 2, it should be emphasized that in the case of foreign bodies, composition, and allergens, caution should be warranted, because when no sampling date is given in the details of the sA notifications, no estimation of a lag phase is possible. In this case, the stakeholders’ (authorities, industry, consumer) performance effectiveness reporting cannot be assessed at all.

With regards to the timely reporting of serious notifications, the fact that the EU RASFF MSs can indeed (to a rather varied degree) notify an sA notification within the 0–10 days lag phase is excellent. More efforts should be made to increase this percent (%) share of occurrence in all hazard categories examined herein, and also to attain a level of homogeneity between the MSs in this particular lag phase. This is truly the most promising finding, since it shows that for metals and microbial contaminants (other), the EU MS have performed exceptionally well by effectively reporting in such a short time. The same was evident for sBR notifications in pesticide residues (much more profoundly though), metals, and microbial contaminants (other).

Mycotoxins, on the other hand, had the smallest relative percent (%) share allocation of both sA and sBR notifications in the 0–10 days lag phase. This represents a true challenge and hence should be the focus of interest in order to increase this in the future. Generally speaking, the aim would have to be to increase the percent share of sA and sBR notifications that are allocated to the shorter, rather than the longer, lag phases. This should hopefully be the overarching goal for all hazard categories examined herein.

Mycotoxins ranked fifth in the frequency of appearance in sA and first in sBR (by far) over the 8-year period examined herein. This finding places mycotoxins ahead of all other hazard categories examined herein in sBR (transgressions originating from third countries). The EU Commission has addressed this plight in European food legislation by implementing a more intensified regulatory control, for example Commission Implementing Regulation (EU) 2019/1793 [14] amended by Commission Implementing Regulation (EU) 2020/625 [15], Commission Implementing Regulation (EU) 2020/1540 [16], and Commission Implementing Regulation (EU) 2021/608 [17].

In a very interesting recent work [7], the authors explored mycotoxins as a case study of particular importance when considering food safety and food security, indicating that mycotoxin contamination can be used as a research lens not only to consider food safety in itself, but also public health more generally, and issues of wider integrity of food supply chains and the impact of a food safety concern on local, national, and global food security.

A lack of harmonization in the allocation of sA and sBR into the different lag phases, represented as a relative percent (%) share, was revealed among the top-three EU RASFF MS for each of the seven or five hazard categories examined, respectively. This raises concerns about the uniformity of a truly integrated/holistic EU approach to the timely reporting of serious food notifications (be it sA or sBR). This lack of harmonization would undoubtedly require an in-depth examination by the European Commission, of course taking into consideration the specific individual state of affairs existing in each MS.

The proposed “lag phases” criterion for sA and sBR notifications in the various hazard categories described in this work could be easily applied to all food hazard categories represented in the RASFF Portal for all years collectively and for a number of years, or on a yearly basis, for all MS (both on an overall and an individual basis), in view of establishing an overall picture of all the EU RASFF MS not only as a whole, but also of every individual MS, thus constructing a respective “RASFF country profile”. The “RASFF country profile” that can be easily constructed for each MS by using this prototype lag phases approach could prove to be quite useful in comparative studies with the overall (all RASFF MS collectively assessed) respective values.

In the context of the Health and Food Audits and Analysis (HFAA, ex-FVO), a country profile is a compilation of key information for each Member State used by the DG Health and Food Safety to support its control and monitoring activities. The FVO country profile includes (i) the five most recently published audit reports, (ii) the Commission’s assessment of the actions taken by the Member States in response to its audits and audit recommendations, (iii) an overview of how controls are organized in the Member States, based on information supplied by them, and (iv) relevant links to Member States’ websites. Each country profile provides a horizontal, country-based overview which (i) facilitates the preparation of audits, (ii) supports and documents the systematic follow-up of recommendations in audit reports, (iii) helps to identify strengths and weaknesses in official controls, and (iv) assists the overall prioritization of audits, and other monitoring activities, and serves as a basic source of background information for stakeholders [18].

In direct accordance with the abovementioned “HFAA country profile”, it is hereby proposed to institutionalize the “RASFF country profile” which would include information on the proposed indicator, namely, “Performance Effectiveness Reporting-50/30 (PER-50/30)” based on the lag phases criterion, and perhaps others to come in the future. The “RASFF country profile” would lend support to official controls enacted by the EU Commission in a uniform way.

This innovative proposal of a performance indicator to gauge each MS’s (and all MS taken as a whole) reporting effectiveness of sA and sBR notifications submission to the EU RASFF aims to conceptualize a means of tangible and quantitative EU MS effectiveness metric that has never been reported in bibliography thus far.

The PER-50/30 indicator has been shown to fluctuate in the different hazard categories examined in sA notifications when all EU MS were included in the exercise. More specifically, metals, microbial contaminants (other), and PMO were the hazard categories that fulfilled this prerequisite, while the other four hazard categories did not, whereas in the case of sBR notifications, this prerequisite was fulfilled in all hazard categories. Thus, it is the sA notifications that present a much greater volatility in terms of this indicator and, as a result of this, would inevitably require a more focused attention for some kind of amelioration towards harmonization on the part of the EU Institutions (e.g., the European Commission).

Nevertheless, in all fairness, depending on the nature of the hazard category (be it physical, chemical, or biological), a horizontally introduced threshold value (e.g., >50%) of a specific lag phase (e.g., 0–30 days) used in this exercise might not be directly applicable, per se, mainly because three hazard categories in sA notifications, namely, composition, foreign bodies, and allergens exhibit a rather high “Subtotal 2”, which does not allow for lag phases to be calculated at all. Perhaps a specific “fit for purpose” threshold value/lag phase combination, ideally specific to each hazard category to apply to all MS, taking into consideration all existing MS parameters (e.g., infrastructure, personnel, funding, national legal framework, etc.), would most probably need to be established. This needs to be further elaborated and thoroughly discussed within the EU Commission and additional research and decision-making processes should be streamlined towards this objective. Necessary legislative action concerning a proposed PER-50/30 indicator should be envisaged.

None of the top-three EU MS in sA notifications in composition, foreign bodies, and allergens could satisfy the effectiveness indicator proposed (PER-50/30) herein, whereas in PMO and microbial contaminants (other), all of the top-three EU MS could. With regards to metals and mycotoxins, only one of the top-three EU MS could satisfy the indicator. Thus, the great fluctuation in performance effectiveness in RASFF reporting, shown in the overall picture, is also shown in the top-three EU MS, and remediation action towards harmonization should be implemented by the EU Commission.

All of the top-three EU MS in sBR notifications in all five hazard categories examined herein satisfied the effectiveness indicator proposed, except for one EU MS in pesticide residues (France) and one EU MS in mycotoxins (the Netherlands). Thus, the volatility in performance effectiveness reporting (as depicted by the PER-50/30 indicator) of the top-three EU MS in sBR is not at all as pronounced as in the sA notifications.

In a report by the Food Chain Evaluation Consortium (FCEC) [19] that was conducted for the European Commission (Directorate-General for Health and Food Safety), it was stated that “Although four in five competent authorities report that they do not use criteria to evaluate the effectiveness of existing crisis management arrangements in their country, most also recognize the utility of having such criteria in place. The definition of harmonized EU criteria to evaluate the effectiveness of existing crisis management arrangements would therefore be welcomed by most competent authorities and other relevant stakeholders”. In the same report, two groups of indicators to evaluate the effectiveness of crisis management arrangements were suggested to be differentiated: process-related indicators and impact-related indicators. Process-related indicators refer to the effectiveness of the processes applied in terms of time needed to detect the outbreak, to identify the source of the outbreak, and to implement related measures until the incident is closed. They do not only refer to crisis management arrangements, but also depend on the effectiveness of surveillance and food control systems in place. Related indicators are (i) the time between the occurrence and detection of the incident, (ii) the time between the detection of the outbreak and the identification of the source, and (iii) the time between the identification of the source and the closure of the incident [19]. Evidently time is of essence in this context, since the longer it takes for an sA or an sBR notification to be submitted in the EU RASFF, the greater the probability that the hazardous food product is potentially distributed in a number of other countries and is thus consumed by a number of people. In principle, the shorter the time from sampling to notification in the EU RASFF is, the more effective the notifying competent authorities are in protecting consumer health.

One should always bear in mind that a comparison between EU RASFF MS was not, nor should it be, per se, the sole objective of this targeted analysis, but rather the revelation of the existing situation within each MS, as well as all EU RASFF MSs, for the selected hazard categories.

A number of EU institutions (Food and Veterinary Office/FVO and the European Commission) have already published reports on the overall operation of official controls performed in Member States, and certain shortcomings have been identified.

A valuable overview report from the FVO [20] was published in order to present a concise analysis of the main findings and conclusions from FVO reports in relation to the performance of laboratories in EU MS with a view to identifying horizontal issues which could usefully be addressed by MS or by the European Commission Services. This report concluded that: “…Notwithstanding severe deficiencies in laboratory capacity and performance in a small number of countries (due principally to alleged budgetary restrictions) it can be said that in general most CAs can have confidence in the general reliability of laboratory performance. This underpins the system for official controls and generally there has been an improving situation over time as regards laboratory performance”.

Generally speaking, one could very justifiably argue that laboratory analysis turnaround times for the analysis of food samples and for the final reporting of results should, at all times, be closely monitored and audited. Additionally, the post-laboratory analysis time period associated with administrative procedures leading to the RASFF notification of the result (being the responsibility of the Competent Authorities) could equally be monitored and audited by the EU Commission (Health and Food Audits and Analysis or the former Food and Veterinary Office), taking under consideration the specific situation (e.g., culture, strategy, planning, facilities, equipment, personnel, financing, etc.) existing in each MS. Furthermore, the Health and Food Audits and Analysis of the European Commission (DG SANTE) could potentially be engaged in a series of targeted audits concerning the turnaround times of food samples analysis in laboratories in the EU MS in view of implementing, for example, the proposed herein PER-50/30 indicator for future use.

Implementation of these proposed “fact-finding” audit series in the EU RASFF MSs, together with the endorsement of a specific or “fit for purpose” PER-50/30 indicator for each hazard category, could pave the way forward, followed by “overview reports” to evaluate the successful implementation of the harmonized approach across the EU MSs.

Best practices identified by the “fact finding” missions in a number of MS (used as successful examples) that have been shown to perform exceptionally well in their task of timely reporting of sA and sBR in the EU RASFF could provide the basis for the harmonization across the board. Mutually acknowledged challenges could emerge, recommendations could be proposed, and solutions could be discussed and agreed upon between the MSs. Clearly, there is still room for improvement for all EU RASFF MSs in view of further enhancing their effectiveness in timely reporting of sA and sBR notifications in the hazard categories on food.

A starting point could be Italy, that in metals, PMO, and microbial contaminants (other) has been shown to have performed quite effectively in the context of timely reporting (high percent shares in the 0–10 days lag phase) of sA notifications to the EU RASFF. Another starting point for sBR notifications, this time, could be Bulgaria, that, in pesticide residues, has also performed very effectively, having submitted just over 80% of all its sBR notifications in the 0–10 days lag phase. In addition, Italy, in metals and microbial contaminants, as well as GB, in PMO, could be seen as example countries that have performed quite well.

Another very interesting report [21] covers the years 2014 to 2016 and concludes that “MS national authorities demonstrated in their annual reports on official controls that they continue to fulfill their important role under the food and feed law. The Commission controls, however, continue to identify also deficiencies in official controls and highlight that there is still room for improvement, and that complacency should be avoided…Nonetheless, the Commission also takes note of the clear signals from MS that staff resources for controls are increasingly limited and that a potential further reduction risks negatively affecting the levels and/or quality of controls and the capacity to respond to emergencies”.

In another important report [22] that covers the years 2017 to 2018, the Commission concludes that “MS national authorities demonstrated in their annual reports on official controls that they continue to fulfill their important role to monitor and to verify, through the organization of official controls, that relevant Union requirements are effectively complied with and enforced. The Commission controls, however, continue to identify deficiencies in official controls and highlight that there is still room for improvement. Relaxed attitudes to food safety always backfire and when they do, it may not only threaten public health but also affect the trust of our citizens as well as our trading partners in the EU food system as a whole. Nevertheless, the Annual Reports for 2017 and 2018 again highlight that many MS are facing challenges to secure adequate financial resources for performing the necessary official controls”.

Recently, an audit by the European Court of Auditors [23] was carried out between December 2017 and May 2018 and concentrated on chemical hazards. The audit question was “Is the EU food safety model soundly based and implemented to keep the products we consume in the EU safe from chemical hazards?”. The audit concluded that the model was soundly based and that it was respected worldwide. However, it was also found that it was currently overstretched, as the Commission and MSs did not have the capacity to implement it fully, and hence challenges were identified regarding its implementation. The Commission accepted the recommendations offered by the audit, namely, (i) reviewing the legislation and improving complementarity between private and public control systems, (ii) maintaining the same level of assurance for both EU-produced and -imported food, and (iii) facilitating consistent application of EU food law.

Other authors [4] have already concluded (from examples described in their study) that “Besides the challenges of timely detection of a hazard also rapid information exchange to relevant parties is also a crucial element. If warnings do not reach the relevant authorities in time, this may hinder timely and adequate prevention, intervention, and control activities. The need of defining sets of criteria to evaluate the performance of early warning and emerging risk identification systems is a prerequisite to justify the investments made”. These authors have argued about the need for criteria, as has the Food Chain Evaluation Consortium or FCEC [19]. The proposals offered herein aim at satisfying this acknowledged requisite for harmonized criteria-based EU evaluation of the effectiveness of the EU RASFF MSs.

The EU Commission has long been engaged in the preparedness and management of crises related to food safety, aiming to avoid or minimize the health and economic impact of possible future crises [24], especially via the Commission Decision (EU) 2019/300 [25] where it is stated that “…Evidence-based, real-time communication to the public and to trade partners is essential to contribute to protecting public health by avoiding further spread of risks…”. This Decision repealed the Commission Decision 2004/478/EC [26] that established a general plan for food crisis management. With regards to risk communication, the flow of information must be timely so that all stakeholders can synchronize their actions accordingly.

To that end, the work herein proposes a novel approach for the EU RASFF, an international Reactive Food Alert and Warning System [27], together with INFOSAN (International Food Safety Authorities Network) [28], the Medical Information System MEDISYS [29], and the World Animal Health Information System (WAHIS) [30].

## 5. Conclusions

An innovative approach was conceptualized, for the first time in bibliography, aiming at introducing a means of gauging the performance effectiveness reporting for the sA and sBR notifications on food, submitted in the EU RASFF until 31 December 2019. The rationale behind this approach was to establish a tangible methodological system of metric assessment with a view to achieving numerical results through the use of a criterion (“lag phases”)-based indicator (PER-50/30). The latter could hopefully be the subject of discussion between MSs and the EU Commission in such a way to be used as “fit for purpose” vis a vis each of the specific hazard categories in the EU RASFF.

The originality of this work is threefold: Firstly, the use of only the alert and border rejection notifications on several hazard categories on food with a serious risk decision from the EU RASFF database. Secondly, the calculation of lag phases (sampling to notification dates as given in the details of each one of the 4503 sA and 5236 sBR notifications) on several hazard categories and their percent (%) share allocation. Thirdly, the introduction of a model Performance Effectiveness Reporting-50/30 (PER-50/30) indicator based on the criterion proposed (lag phases) for all hazard categories examined herein.

This prototype proposal to the EU Commission (DG SANTE) for the actual measurement of the MSs’ performance effectiveness in their timely contribution of sA and sBR notifications to the EU RASFF could be envisioned to be implemented in the following actions: (i) construction of “RASFF country profiles” in direct accordance to the “FVO country profiles” to be used in a complementary mode, (ii) implementation of fact-finding missions and resulting overview reports for all MSs based on the indicator proposed, and (iii) establishment of greater degrees of harmonization of the RASFF EU MSs’ performance effectiveness in reporting sA and sBR to the EU RASFF database.

## Figures and Tables

**Table 1:** Yearly distribution of serious alert (sA) notifications, in seven hazard categories (mycotoxins, microbial contaminants (other), foreign bodies, composition, allergens, metals, pathogenic microorganisms (PMO)) and their sum as a percent share (%) of all sA in all hazard categories in the EU RASFF database (2012–2019).

Year	Numbers of sA Notifications on Food in Seven Hazard Categories and Percent Share of Each One over All Hazard Categories							
	Metals	Microbial Contaminants (Other)	Mycotoxins	PMO	Composition	Foreign Bodies	Allergens	Sum of Notifications/All sA Notifications in All Hazard Categories (%)
2012	16	29	14	41	26	14	25	165/210 (78.6%)
2013	63	79	64	123	18	27	42	416/520 (80.0%)
2014	87	85	46	136	52	34	56	496/635 (78.1%)
2015	66	87	70	158	36	42	105	564/664 (84.9%)
2016	64	102	78	128	63	70	84	589/722 (81.6%)
2017	104	79	68	200	50	74	113	688/815 (84.4%)
2018	53	117	84	219	77	89	136	775/937 (82.7%)
2019	60	130	60	180	122	88	170	810/977 (82.9%)
Sum	513	708	484	1185	444	438	731	4503/5480 (82.1%)

**Table 2:** Serious alert (sA) notifications on the seven food hazard categories (mycotoxins, microbial contaminants (other), foreign bodies, composition, allergens, metals, pathogenic microorganisms (PMO)) submitted by all EU RASFF MS (overall) on the basis of BCCD, BCCR, COC, CC, FP, MoM, OCnonMS, and OCTM.

	Percent (%) Share of sA Notifications Made on the Basis of							
Hazard Categories	BCCD	BCCR	COC	CC	FP	MoM	OCnonMS	OCTM
Metals	0.2	5.8	10.1	0.6	0.0	0.0	0.0	83.2
Microbial Contaminants (other)	0.0	1.5	53.7	0.6	2.5	0.0	0.7	41.0
Mycotoxins	0.2	5.8	21.3	0.2	0.0	0.0	0.4	72.1
PMO	0.0	1.9	48.3	0.6	12.3	0.0	0.4	36.5
Composition	0.0	2.2	2.7	4.9	1.3	15.8	0.2	72.7
Foreign Bodies	0.0	0.0	32.4	63.5	0.0	0.0	0.0	4.1
Allergens	0.0	0.3	47.5	10.3	5.6	0.0	0.4	36.00

BCCD = border control—consignment detained, BCCR = border control—consignment released, COC = company’s own check, CC = consumer complaint, FP = food poisoning, MoM = monitoring of media, OCnonMS = official control in non-member state, OCTM = official control on the market.

**Table 3:** Percent (%) share of lag phases in serious alert (sA) notifications of the seven food hazard categories (mycotoxins, microbial contaminants (other), foreign bodies, composition, allergens, metals, PMO) for all EU RASFF MS collectively, originating from the EU RASFF database (2012–2019).

	Percent (%) Share of sA Notifications (% of Sum of Each Hazard Category)						
Lag Phases (Days)	Metals (%)	Microbial Contaminants (Other) (%)	Mycotoxins (%)	PMO (%)	Composition (%)	Foreign Bodies (%)	Allergens (%)
0–10	31.6	43.8	9.5	26.4	7.2	12.5	8.2
11–20	23.4	31.1	14.9	31.7	6.1	3.0	7.47
21–30	14.8	10.0	15.7	13.6	7.6	0.7	6.7
31–40	8.6	2.1	12.4	4.9	7.6	0.4	5.3
41–50	5.6	1.1	13.0	2.0	3.6	0.2	2.9
51–100	8.8	1.5	24.4	3.3	23.6	0.4	8.2
≥101	5.3	0.4	7.2	1.8	11.7	0.0	1.9
Subtotal 1 *	98.0	90.1	97.1	83.7	67.6	17.3	40.6
No sampling date	1.4	9.6	2.3	15.4	30.6	82.6	59.4
Inverse dates	0.4	0.1	0.6	0.7	1.8	0.0	0.0
Dated 1/1/1970	0.2	0.1	0.0	0.2	0.0	0.0	0.0
Subtotal 2 **	1.9	9.9	2.9	16.3	32.4	82.6	59.4

Subtotal 1 * = sum of percent (%) share of sA without shortcomings; Subtotal 2 ** = sum of percent (%) share of sA with shortcomings.

**Table 4:** Percent (%) share of lag phases in serious alert (sA) notifications in the top-three EU RASFF MS in each of the seven hazard categories (mycotoxins, microbial contaminants (other), foreign bodies, composition, allergens, metals, pathogenic microorganisms (PMO)), according to the EU RASFF database (2012–2019).

Lag Phase (Days)	Percent (%) Share of sA Notifications in Lag Phases in the Top-Three EU RASFF MS in Seven Hazard Categories																				
	Metals (%)			Micr. Contam. (Other) (%)			Mycotoxins (%)			PMO (%)			Composition (%)			Foreign Bodies (%)			Allergens (%)		
	IT	NL	DE	FR	IT	BE	DE	NL	BE	FR	NL	IT	DE	SE	FR	DE	GB	NL	GB	NL	BE
0–10	49.5	5.1	27.6	47.6	54.4	44.4	6.7	0.0	30.2	26.5	15.9	35.4	0.8	31.1	0.0	21.0	0.0	0.0	7.5	9.2	4.4
11–20	26.7	25.6	3.4	33.5	25.6	36.1	9.2	12.1	39.6	29.7	22.5	30.0	5.9	6.5	5.5	4.2	0.0	0.0	2.5	2.0	7.3
21–30	12.6	7.7	13.8	7.8	10.4	11.1	14.2	7.6	15.1	12.4	13.9	10.8	8.5	3.3	5.5	0.0	0.0	0.0	0.0	1.0	2.9
31–40	5.6	7.7	3.4	1.4	1.6	1.4	10.8	3.0	7.5	8.4	1.3	4.6	11.0	0.0	0.0	0.0	0.0	0.0	2.5	0.0	2.9
41–50	2.4	7.7	10.3	1.4	0.8	0.0	17.5	6.1	0.0	4.4	4.0974	0.0	6.8	0.0	5.5	0.7	0.0	0.0	2.5	1.0	0.0
51–100	2.4	20.5	20.7	2.9	0.0	0.0	37.5	36.4	3.8	3.6	2.6	1.5	34.7	6.5	36.1	0.7	0.0	0.0	4.2	1.0	0.0
≥101	0.3	20.5	20.7	0.5	0.0	0.0	2.5	33.3	0.0	1.2	4.0	0.0	18.6	0.0	19.4	0.0	0.0	0.0	0.0	2.0	0.0
Subtotal 1 *	99.6	94.9	100.0	95.1	92.8	93.0	98.3	98.5	96.2	86.3	64.2	82.3	86.4	47.5	72.2	26.6	0.0	0.0	19.2	16.3	17.6
No sampling date	0.3	5.1	0.0	4.4	7.2	6.9	0.0	1.5	3.8	12.8	34.4	17.7	11.0	50.8	25.0	73.4	97.9	100.0	80.8	83.7	82.3
Inverse dates	0.0	0.0	0.0	0.5	0.0	0.0	1.7	0.0	0.0	0.8	1.3	0.0	2.5	1.6	2.8	0.0	2.0	0.0	0.0	0.0	0.0
Dated 1/1/1970	0.0	0.0	0.0	0.0	0.0	0.0	0.0	0.0	0.0	0.0	0.0	0.0	0.0	0.0	0.0	0.0	0.0	0.0	0.0	0.0	0.0
Subtotal 2 **	0.3	5.1	0.0	4.8	7.2	6.9	1.7	1.5	3.8	13.6	35.8	17.7	13.5	52.4	27.8	73.4	100.0	100.0	80.8	83.7	82.3

Subtotal 1 * = sum of percent (%) share of sA without shortcomings; Subtotal 2 ** = sum of percent (%) share of sA with shortcomings.

**Table 5:** Serious alert (sA) notifications on the seven food hazard categories (mycotoxins, microbial contaminants (other), foreign bodies, composition, allergens, metals, PMO) submitted by the top-three EU RASFF MS on the basis of BCCD, BCCR, COC, CC, FP, MoM, OCnonMS, and OCTM.

Hazard Category	Percent (%) Share of sA Notifications Submitted to RASFF on the Basis of							
Metals	BCCD	BCCR	COC	CC	FP	MoM	OCnonMS	OCTM
IT	0.0	2.1	3.9	0.3	0.0	0.0	0.0	93.7
NL	0.0	0.0	33.3	0.0	0.0	0.0	0.0	66.7
DE	0.0	38.0	13.8	3.4	0.0	0.0	0.0	44.8
Microbial Contaminants (other)								
FR	0.0	0.0	85.4	0.0	2.4	0.0	0.5	11.6
IT	0.0	0.8	12.8	0.0	2.4	0.0	1.6	82.4
BE	0.0	0.0	61.1	1.4	0.0	0.0	0.0	37.5
Mycotoxins								
DE	0.0	1.7	15.0	0.0	0.0	0.0	0.0	83.3
NL	0.0	0.0	18.2	0.0	0.0	0.0	1.5	80.3
BE	1.9	22.6	54.7	0.0	0.0	0.0	0.0	20.7
PMO								
FR	0.0	0.0	67.5	0.4	19.3	0.0	0.0	12.8
NL	0.0	6.0	58.3	0.0	7.9	0.0	0.0	27.8
IT	0.0	0.0	7.0	1.5	13.8	0.0	0.0	77.7
Composition								
DE	0.0	0.0	0.8	7.6	0.8	6.8	0.0	83.9
SE	0.0	0.0	0.0	6.6	0.0	64.0	0.0	29.5
FR	0.0	0.0	0.0	0.0	2.8	5.6	0.0	91.7
Foreign Bodies								
DE	0.0	0.0	25.2	70.6	0.0	0.0	0.0	4.2
GB	0.0	0.0	49.0	44.9	0.0	0.0	0.0	6.1
NL	0.0	0.0	74.4	25.6	0.0	0.0	0.0	0.0
Allergens								
GB	0.0	0.0	51.7	6.7	3.3	0.0	0.0	38.3
NL	0.0	0.0	84.7	10.2	2.0	0.0	0.0	3.1
BE	0.0	0.0	80.9	5.9	1.5	0.0	0.0	11.8

BCCD = border control—consignment detained, BCCR = border control—consignment released, COC = company’s own check, CC = consumer complaint, FP = food poisoning, MoM = monitoring of media, OCnonMS = official control in non-member state, OCTM = official control on the market.

**Table 6:** Yearly distribution of serious border rejection (sBR) notifications in five hazard categories (mycotoxins, microbial contaminants (other), pesticide residues, metals, pathogenic microorganisms (PMO)), and their sum as a percent share (%) of all sBR notifications in all hazard categories in the EU RASFF (2011–2019).

Year	Numbers of sBR Notifications on Food in Five Hazard Categories and Percent Share of Each One over All Hazard Categories					
	Metals	Microbial Contaminants (Other)	Mycotoxins	PMO	Pesticide Residues	Sum of Notifications/All sBR Notifications in All Hazard Categories (%)
2011	0	0	0	1	0	1/1 (100.0%)
2012	9	12	160	34	27	242/278 (87.0%)
2013	20	59	259	109	48	495/542 (91.3%)
2014	22	14	270	149	58	513/591 (86.8%)
2015	20	3	375	203	53	654/682 (95.9%)
2016	22	8	408	122	90	650/696 (93.4%)
2017	32	27	445	376	99	979/1018 (96.2%)
2018	12	23	457	242	108	842/885 (95.1%)
2019	10	10	408	298	134	860/900 (95.5%)
Sum	147	156	2782	1534	617	5236/5593 (93.6%)

**Table 7:** Percent (%) share of lag phases in sBR notifications of the five food hazard categories (mycotoxins, microbial contaminants (other), pesticide residues, metals, pathogenic microorganisms (PMO)) for all EU RASFF MS collectively, originating from the EU RASFF database (2011–2019).

	Percent (%) Share of sBR Notifications in Lag Phases in Five Hazard Categories				
Lag Phases (Days)	Metals (%)	Microbial Contaminants (Other) (%)	Mycotoxins (%)	PMO (%)	Pesticide Residues (%)
0–10	36.7	30.1	16.6	23.4	60.4
11–20	36.7	23.7	28.4	43.9	13.8
21–30	8.2	15.4	18.3	14.8	6.5
31–40	8.8	11.5	9.6	5.5	4.5
41–50	2.0	6.4	6.9	3.5	4.4
51–100	4.1	1.9	12.7	4.6	5.2
≥101	1.3	0.6	5.3	2.1	2.3
Subtotal 1 *	97.9	89.7	97.8	97.8	97.1
No sampling date	1.4	9.6	1.4	1.7	1.4
Inverse dates	0.7	0.0	0.6	0.4	1.4
Dated 1/1/1970	0.0	0.6	0.2	0.1	0.0
Subtotal 2 **	2.0	10.2	2.2	2.1	2.9

Subtotal 1 * = sum of percent (%) share of sBR without shortcomings; Subtotal 2 ** = sum of percent (%) share of sBR with shortcomings.

**Table 8:** Percent (%) share of lag phases in serious border rejection (sBR) notifications in the top-three EU RASFF MSs in each of the five food hazard categories (mycotoxins, microbial contaminants (other), pesticide residues, metals, PMO), according to the EU RASFF database (2011–2019).

	Percent (%) Share of sBR Notifications in Lag Phases in the Top-Three EU RASFF MS														
	Top-Three EU MS (in Notification Numbers)														
	Metals (%)			Micr. Contam. (Other) (%)			Mycotoxins (%)			PMO (%)			Pesticide Residues (%)		
Lag Phase (Days)	FR	IT	ES	NL	IT	DE	NL	DE	GB	NL	GB	GR	BG	GB	FR
0–10	39.5	41.7	19.0	9.1	50.0	52.9	9.6	11.4	22.7	18.7	48.7	9.8	82.3	45.4	4.4
11–20	39.5	44.4	52.4	34.8	26.9	29.4	20.0	31.4	34.5	50.8	33.1	45.4	6.1	26.1	15.5
21–30	10.5	2.8	14.3	22.7	7.7	0.0	16.0	23.6	15.1	14.8	7.6	18.5	4.2	5.7	15.5
31–40	2.6	5.5	14.3	15.1	0.0	5.9	8.6	10.7	8.6	4.4	3.5	6.2	1.3	3.4	13.3
41–50	0.0	2.8	0.0	12.1	0.0	0.0	9.0	7.9	1.8	4.4	1.2	5.1	2.9	4.5	11.1
51–100	2.6	0.0	0.0	1.5	0.0	0.0	20.0	9.5	9.0	3.4	2.6	8.0	0.6	7.9	17.8
≥101	2.6	2.8	0.0	0.0	3.8	0.0	15.6	3.9	4.7	1.5	1.5	5.8	1.0	3.4	13.3
Subtotal 1 *	97.4	100.0	100.0	95.4	88.5	88.2	99.0	98.4	96.4	98.0	98.2	98.9	98.4	96.6	91.1
No sampling date	2.6	0.0	0.0	4.5	11.5	11.8	0.4	1.1	1.8	1.7	1.5	1.1	1.3	3.4	0.0
Inverse dates	0.0	0.0	0.0	0.0	0.0	0.0	0.6	0.2	1.4	0.2	0.3	0.0	0.3	0.0	8.9
Dated 1/1/1970	0.0	0.0	0.0	0.0	0.0	0.0	0.0	0.2	0.4	0.0	0.0	0.0	0.0	0.0	0.0
Subtotal 2 **	2.6	0.0	0.0	4.5	11.5	11.8	1.0	1.6	3.6	1.9	1.8	1.1	1.6	3.4	8.9

Subtotal 1 * = sum of percent (%) share of sBR without shortcomings; Subtotal 2 ** = sum of percent (%) share of sBR with shortcomings.

## Data Availability

The data presented in this study are openly available in the European Commission RASFF portal database (https://webgate.ec.europa.eu/rasff-window/portal, (accessed on 16 May 2020)).
